# PIM1 orchestrates sepsis-associated inflammatory imbalance in CD4^+^ T cell subsets via cholesterol metabolism

**DOI:** 10.1128/mbio.01680-25

**Published:** 2025-09-03

**Authors:** Mei Liu, Jia-Qi Wang, Jia-Ning Wang, Yi-Ni Sun, Si-Yao Li, Ya-Jing Fu, Yong-Jun Jiang, Zi-Ning Zhang, Hong Shang

**Affiliations:** 1 National Clinical Research Center for Laboratory Medicine, The First Hospital of China Medical University 159407, Shenyang, China; 2 State Key Laboratory for Diagnosis and Treatment of Infectious Diseases, NHC Key Laboratory of AIDS Prevention and Treatment, National Clinical Research Center for Laboratory Medicine, The First Hospital of China Medical University, China Medical University, Shenyang, China; 3 Key Laboratory of AIDS Immunology, Chinese Academy of Sciences 53037 , Shenyang, China; 4 Key Laboratory of AIDS Immunology of Liaoning Province, Shenyang, China; 5 Department of Critical Care Medicine, The First Hospital of China Medical University 159407, Shenyang, China; Monash University, Clayton, Victoria, Australia

**Keywords:** sepsis, PIM1, CD4^+ ^T cell, inflammation, cholesterol metabolism

## Abstract

Sepsis, caused by an unbalanced host response to infection, remains a global health burden. The dysregulation between pro-inflammatory and anti-inflammatory responses is a primary driver of immune imbalance. As a central player in adaptive immunity, CD4^+^ T cells are crucial for maintaining this balance during sepsis by differentiating into various effector T cell subsets. Thus, elucidating the underlying mechanisms within the inflammatory and anti-inflammatory imbalance in CD4^+^ T cell subsets during sepsis is of great value. We first identified the proviral integration site for Moloney murine leukemia virus 1 (PIM1) as a significantly upregulated gene in CD4^+^ T cells from sepsis patients by conducting a comprehensive transcriptome meta-analysis. The expression of PIM1 was significantly elevated on CD4^+^ T cells from sepsis patients and was correlated with both SOFA and APACHE II scores. Moreover, we found that PIM1 promoted the differentiation of CD4^+^IFN-γ^+^ Th1 and CD4^+^IL-17A^+^ Th17 subsets while inhibiting the differentiation of CD25^+^FoxP3^+^ Tregs. Additionally, upon inhibiting PIM1 kinase activity in CD4^+^ T cells, RNA sequencing analysis revealed that the cholesterol metabolism gene, ATP-binding cassette transporter G1 (ABCG1), exhibited significant upregulation. More importantly, we found that the intracellular cholesterol content was decreased in CD4^+^ T cells after inhibiting the PIM1 kinase activity or knocking down PIM1. The portion of CD4^+^IFN-γ^+^ Th1 and CD4^+^IL-17A^+^ Th17 cells was recovered, and the CD4^+^CD25^+^FoxP3^+^ Tregs decreased after cholesterol supplementation to CD4^+^ T cells. These findings indicated that PIM1 may regulate the balance of Th1, Th17, and Treg subsets in a cholesterol-dependent manner in sepsis.

**IMPORTANCE:**

This study aims to elucidate the mechanism of the inflammatory and anti-inflammatory imbalance of CD4^+^ T cell subsets during sepsis. Our study provides evidence that PIM1 serves as a crucial regulator of sepsis-induced inflammation and elucidates that PIM1 participates in regulating the imbalance of Th1, Th17, and Treg subsets, further promoting inflammatory and anti-inflammatory imbalance in sepsis. Additionally, the cholesterol metabolism, potentially mediated by ABCG1, is implicated in PIM1’s regulatory effect on the Th1, Th17, and Treg imbalance. Our study provides novel insights into the inflammatory imbalance during sepsis, which could facilitate the development of therapeutic strategies aimed at modulating the immune-inflammatory cascade in this condition.

## INTRODUCTION

Sepsis is defined as life-threatening organ dysfunction due to a dysregulated host response to infection (1). Sepsis is a devastating healthcare problem all over the world with a high mortality rate and proven economic burden (2, 3). Although the pathological mechanisms of sepsis remain to be fully elucidated, it has been confirmed that immune system dysfunction is the main pathophysiological process in the development of sepsis (4). The dysregulation between pro-inflammatory and anti-inflammatory responses is a major cause of exaggerated inflammatory responses and cytokine storm, consequently resulting in multiple organ dysfunction syndrome (MODS) and even death in patients with sepsis (5). Therefore, there is an urgent need to further explore the imbalance of pro-inflammatory and anti-inflammatory responses in sepsis to better control the disease.

Beyond innate immunity, researchers have demonstrated that adaptive immunity also exerts a substantial influence on the inflammatory imbalance observed in sepsis (6–8). As a central player in adaptive immunity, CD4^+^ T cells play a vital role in maintaining the balance of pro-inflammatory and anti-inflammatory responses and the progression of sepsis by differentiating into distinct types of effector T cells (9, 10). Th1 and Th17 cells, distinguished by their production of the hallmark cytokines interferon (IFN)-γ and interleukin (IL)−17, respectively, are primary pro-inflammatory CD4^+^ T cell subsets in sepsis (11). In contrast, regulatory T cells (Tregs), characterized by the expression of Forkhead box protein 3 (Foxp3), serve as the main anti-inflammatory counterparts by secreting IL-10 and transforming growth factor (TGF)-β to maintain immune homeostasis (12). The delicate balance among Th1, Th17, and Tregs is considered crucial for modulating the pro-inflammatory and anti-inflammatory responses in sepsis. Patients with post-traumatic conditions who had developed sepsis exhibited a disruption in cell-mediated immune responses and an imbalance among Th1, Th17, and Treg populations, along with other T helper subsets (9). Liu et al. (13) confirmed that the proportion of Th17 cells was significantly increased in sepsis patients, which was closely related to the severity of the disease, increased levels of inflammation, and poor prognosis. Treg cells show an increased proportion in the peripheral blood of sepsis patients, which affects the degree of immune imbalance and the prognosis of the disease (14). In addition, Zhou et al. (15) found that patients who develop sepsis-induced acute kidney injury (SAKI) exhibit higher Th17/Treg ratio levels compared with patients without renal injury, indicating that factors that promote the differentiation of Th17 cells while simultaneously inhibiting Treg cells may be correlated with disease progression and MODS. The changes in CD4^+^ T cell subsets might differ between patients with sepsis caused by different primary diseases or at different stages. Elucidating the underlying mechanisms within the inflammatory and anti-inflammatory imbalance of CD4^+^ T cell subsets during sepsis is of great value.

More and more evidence has shown that cellular metabolism plays a pivotal role in the pathogenesis of various inflammatory diseases by regulating T-cell activation and differentiation (16, 17). CD4^+^ T cell subsets exhibit a fundamental difference in metabolic requirements and the effect of manipulating metabolic pathways. Studies have demonstrated that the effector T cell populations, including Th1, Th2, and Th17, cells exhibit enhanced glycolysis but less oxidative phosphorylation (OXPHOS) than in Tregs that utilize lipid peroxidation (LPO) or fatty acid oxidation (FAO) and OXPHOS (18). The pathogenesis of sepsis involves alterations in immune cell metabolism, which play a crucial role in modulating immune function during this severe inflammatory disease (19). T cells derived from the septic patients exhibited a significantly defective OXPHOS, glucose metabolism, and lipid metabolism (18, 20). In recent years, studies have emphasized the significant role that cholesterol metabolism plays in modulating T-cell activation, differentiation, and function. The cholesterol metabolism acts as a significant regulatory “switcher” of CD4^+^ T cells in inflammatory response (21–23). The alterations in CD4^+^ T cell subsets caused by dysregulated cholesterol metabolism have been reported to participate in the pathogenesis of various inflammatory-related diseases, including autoimmune diseases, atherosclerosis, and intestinal inflammation, etc. (24–26). Nevertheless, the changes in cholesterol metabolism within CD4^+^ T cells during sepsis and their subsequent effects on the differentiation of CD4^+^ T cell subsets have yet to be fully elucidated.

In the present study, we conducted a comprehensive transcriptomic meta-analysis, uncovering a significant upregulation of proviral integration site for Moloney murine leukemia virus 1 (PIM1) in CD4^+^ T cells from patients with sepsis. A strong positive correlation was identified between the levels of PIM1 expression and the severity of sepsis. Notably, inhibition of PIM1 led to a reduction in the proportions of IFN-γ-producing Th1 and IL-17-producing Th17 cells while simultaneously inducing the expression of CD4^+^CD25^+^Foxp3^+^ Treg cells, thereby contributing to the imbalance among Th1, Th17, and Treg subsets. Additionally, our research confirmed that PIM1 promotes this imbalance by decreasing intracellular cholesterol content, thereby providing further insights into the molecular mechanisms underlying the dysregulation of inflammatory and anti-inflammatory responses in sepsis.

## MATERIALS AND METHODS

### Study population and recruitment

Patients admitted to the Department of Intensive Care Medicine and the Department of Infectious Diseases of The First Hospital of China Medical University were enrolled. Sepsis was diagnosed according to the guidelines from The Third International Consensus Definitions for Sepsis and Septic Shock (Sepsis-3) (1). All participants were aged 18 years or older. Exclusion criteria included a history of chemoradiotherapy or immunotherapy within the preceding 6 months, as well as diagnoses of autoimmune diseases, tumors, or active HIV/HBV/HCV infections. A total of 67 sepsis patients and 74 age- and sex-matched healthy controls were included in the study. The baseline characteristics and clinical parameters of septic patients are presented in Tables S1 and S2.

### Transcriptome meta-analysis

The transcriptional profiles of whole blood CD4^+^ T cells in sepsis patients (GSE133822 and GSE136200) were downloaded from the GEO database. The transcriptional meta-analysis was performed by the integrative meta-analysis of expression data (INMEX) program (https://www.networkanalyst.ca/). After the log2 transformation, the intensity values for each probe were uploaded, processed, and annotated to ensure integrity. Following a comprehensive data integrity check, we utilized a combined *P* value method, a standard approach in the meta-analysis of microarray data, to identify the differentially expressed genes (DEGs). Genes with a combined *P* value below the 0.05 threshold were considered to be DEGs. Gene ontology (GO) and Kyoto Encyclopedia of Gene and Genome (KEGG)-enriched pathways were identified using KOBAS version 3.0 (http://bioinfo.org/kobas) to identify the significant pathways of CD4^+^ T cells during sepsis.

### Evaluating cell viability following AZD1208 exposure

In total, 200 µL of cell suspension at a density of 1 × 10⁶ cells/mL was seeded into 96-well plates. Then, a range of AZD1208 (MedChem Express, China) concentrations (2.5 µM, 5 µM, 10 µM, 20 µM, and 40 µM) was introduced into each well, respectively, followed by the addition of anti-CD3/CD28 beads (Gibco Technologies, USA) to activate the cells. Cells were incubated for 24 h at 37°C in a 5% CO₂ atmosphere, and 20 µL of CCK-8 (DOJINDO, Japan) solution was added to each well 3 h in advance. Absorbance was detected at 450 nm using a microplate reader. The values were normalized to the absorbance of blank wells, and the relative cell viability was expressed as a percentage relative to dimethyl sulfoxide (DMSO)-treated control wells.

### siRNA transfection

Peripheral blood mononuclear cells (PBMCs) from healthy donors were isolated by density gradient centrifugation using Ficoll-Paque PLUS (GE Healthcare Life Sciences, USA). CD3^+^ T cells were isolated from PBMCs by the EasySep Human T Cell Isolation Kit (STEMCELL Technologies, Canada). A total of 3 × 10^6^ to 5 × 10^6^ CD3^+^ T cells were directly electroporated with 2 nmol/sample Stealth siRNAs for PIM1 (Thermo Fisher Scientific, USA) or 20 pmol/sample Silencer Select siRNAs for ABCG1 (Thermo Fisher Scientific, USA) using Human T cell Nucleofector Solution (LONZA Amaxa) under the U-14 program (LONZA Amaxa). After transfection for 24 h, the cells were harvested for the following experiments. The knockdown efficiency of siRNA transfection was examined using real-time PCR. The cell viability was evaluated using Live/Dead dye staining (Thermo Fisher Scientific, USA) to exclude dead cells prior to flow cytometry analysis.

### Flow cytometry analysis

CD3^+^ T and CD4^+^ T cells were separated from PBMCs by the EasySep Human T Cell Isolation Kit and EasySep Human CD4^+^ T Cell Isolation Kit (STEMCELL Technologies, Canada), respectively. Following viability staining with the Live/Dead dye (Thermo Fisher Scientific, USA), the cells were harvested and stained with surface marker antibodies, including PerCP anti-human CD3 antibody (BioLegend, USA), APC/Cyanine7 anti-human CD4 antibody (BioLegend, USA), and PE anti-human CD25 antibody (BioLegend, USA). For PD-1 and TIM-3 staining, the cells were harvested and stained with surface marker antibodies, PerCP anti-human CD8 antibody (BioLegend, USA), APC/Cyanine7 anti-human CD4 antibody (BioLegend, USA), BV421 anti-human CD279 antibody (BioLegend, USA), and PE anti-human TIM-3 antibody (BioLegend, USA).

For intracellular IL-17A and IL-22 staining, the isolated cells were cultured in RPMI Media 1640 (Life Technologies, Thermo Fisher Scientific, USA) supplemented with 10% FBS and 1% PS. Cells were stimulated with eBioscience Cell Stimulation Cocktail, a mixture containing phorbol 12-myristate 13-acetate (PMA), ionomycin, brefeldin A, and monensin (Invitrogen, USA) for 4 h at 37°C to stimulate cytokine production. After stimulation, the harvested cells were fixed, permeabilized, and stained with APC anti-human IL-17A (Biolegend, USA) or FITC anti-human IL-22 (BioLegend, USA). For IFN-γ staining, the harvested CD3^+^ T cells were stimulated with Dynabeads Human T-Activator CD3/CD28 (Gibco Technologies, USA) at a bead-to-cell ratio of 1:2 at 37°C for 24 h. After stimulation, the harvested cells were fixed, permeabilized, and stained with BV421 anti-human IFN-γ (BioLegend, USA). For granzyme B staining, the harvested cells were fixed, permeabilized, and stained with BV421 anti-human Granzyme B (BioLegend, USA).

For intracellular transcription factor staining, the harvested CD3^+^ T cells were stimulated with Dynabeads Human T-Activator CD3/CD28 (Gibco Technologies, USA) at a bead-to-cell ratio of 1:2 at 37°C for 24 h. After the surface staining, the cells were fixed and permeabilized with the eBioscience Fixation/Permeabilization kit (Invitrogen, USA) following the manufacturer’s instructions. The cells were stained for 1 h at 4°C with FITC anti-human FoxP3 antibody (BioLegend, USA) and washed twice with the permeabilization buffer. For Ki-67 staining, the isolated cells were fixed and permeabilized and stained for 1 h at 4°C with BV421 anti-human Ki-67 antibody (BioLegend, USA) and washed twice with the permeabilization buffer.

For intracellular staining for PIM1, the cells were fixed, permeabilized, and then blocked with 5% BSA. After blocking, the cells were incubated with anti-PIM1 antibody (1:200, NOVUS, USA) for 1 h at 4°C, followed by incubation with the Goat anti-Rabbit IgG (H + L) conjugated with Alexa Fluor Plus 488 secondary antibody (Thermo Fisher Scientific, USA). After being washed using PBS, the cells were analyzed using the FACSCanto II flow cytometer (BD Biosciences, USA), and the percentages of subsets and MFI were analyzed using FlowJo software version 10.4 (BD Biosciences, USA).

To analyze the intracellular cholesterol level of the CD4^+^ T cells and PBMCs, the harvested cells were stained with the PerCP anti-human CD3 antibody (BioLegend, USA) and APC/Cyanine7 anti-human CD4 (BioLegend, USA) antibodies. After fixation using 4% paraformaldehyde fix solution for 15 min, the cells were washed twice at room temperature (RT) and stained with 50 µg/mL filipin III (Sigma-Aldrich, USA) for 50 min at RT. After washing twice with PBS, the cells were analyzed by flow cytometry immediately. The MFI of filipin III on PBMC and CD4^+^ T cells was assessed on the FACSCanto II flow cytometer (BD Biosciences, USA). To analyze the T cell lipid raft, following viability staining with the Live/Dead dye (Thermo Fisher Scientific, USA), the harvested T cells were stained with the PerCP anti-human CD3 antibody (BioLegend, USA) and APC/Cyanine7 anti-human CD4 (BioLegend, USA) antibodies. Cells were then washed and stained with 500 ng/mL choleratoxin B-AF488 (Invitrogen, USA) for 1 h at RT. Choleratoxin B fluorescence on CD4^+^ T cells was assessed on the FACSCanto II flow cytometer (BD Biosciences, USA). The FACS data were analyzed using the FlowJo software version 10.4.

### Real-time PCR

The cells were collected and washed with PBS, and total RNA from T cells and PBMCs was extracted using the RNeasy Mini Kit (QIAGEN, USA). The cDNA was synthesized with the PrimeScript RT reagent kit, following the two-step procedure (Takara, Japan). Real-time quantitative PCR was conducted with TB Green Master Mix (Takara, Japan) on Roche LightCycler 480 (Roche, USA). The fold change in gene expression was determined by employing the ΔΔCt method, with Gapdh serving as the internal reference gene. Primers used in this study are the following: GAPDH Forward, AATGACCCCTTCATTGAC, and Reverse, TCCACGACGTACTCAGCGC; PIM1 Forward, CGAGCATGACGAAGAGATCAT, and Reverse, TCGAAGGTTGGCCTATCTGA; ABCG1 Forward, AATAACCTCACGGAAGCCCA, and Reverse, CAGCCAGGATGTTCATCAGC; LXRβ Forward, CTCTCCTACCACGAGTTCCC, and Reverse, CCTCTTCGGGATCTGGGATG; SREBP2 Forward, CTCACCTTCCTGTGCCTCTC, and Reverse, AGGCATCATCCAGTCAAACC; SCAP Forward, CGCAAACAAGGAGAGCCTAC, and Reverse, TGTCTCTCAGCACGTGGTTC; and ACAT1 Forward, TTCAGGGAGCCATTGAAAAG, and Reverse, GGCTTTCATTCCTGAAGCAC.

### RNA-seq analysis

CD4^+^ T cells were isolated from PBMCs via magnetic separation using the human CD4^+^ T Cell Isolation Kit. CD4^+^ T cells were activated by CD3/CD28 Dynabeads at a bead-to-cell ratio of 1:2 for 24 h. The PIM kinase inhibitor AZD1208 was added to the medium, with DMSO serving as the control. The process of cDNA library construction, purification, and RNA sequencing (RNA-seq) was executed on the BGISEQ platform developed by Huada Gene Technology (Shenzhen, China). Standard bioinformatics analysis was then performed by utilizing the BGI online system (https://biosys.bgi.com), accessible through their official website.

### Statistics

Statistical analysis and data presentation were performed by GraphPad Prism v 9.0. The results are presented as the mean ± SEM. The statistical significance was calculated using the Mann-Whitney *U* test between unpaired groups and the Wilcoxon signed-rank test between the paired groups. The data presented were representative of at least three independent experiments. Spearman’s correlation analysis was applied to perform the correlation analysis. *P* < 0.05 was considered to be statistically significant (*P < 0.05; **P < 0.01; ***P < 0.001; and ****P < 0.0001).

## RESULTS

### PIM1 expression is altered in sepsis-associated CD4^+^ T cells by transcriptome meta-analysis

**Fig 1:**
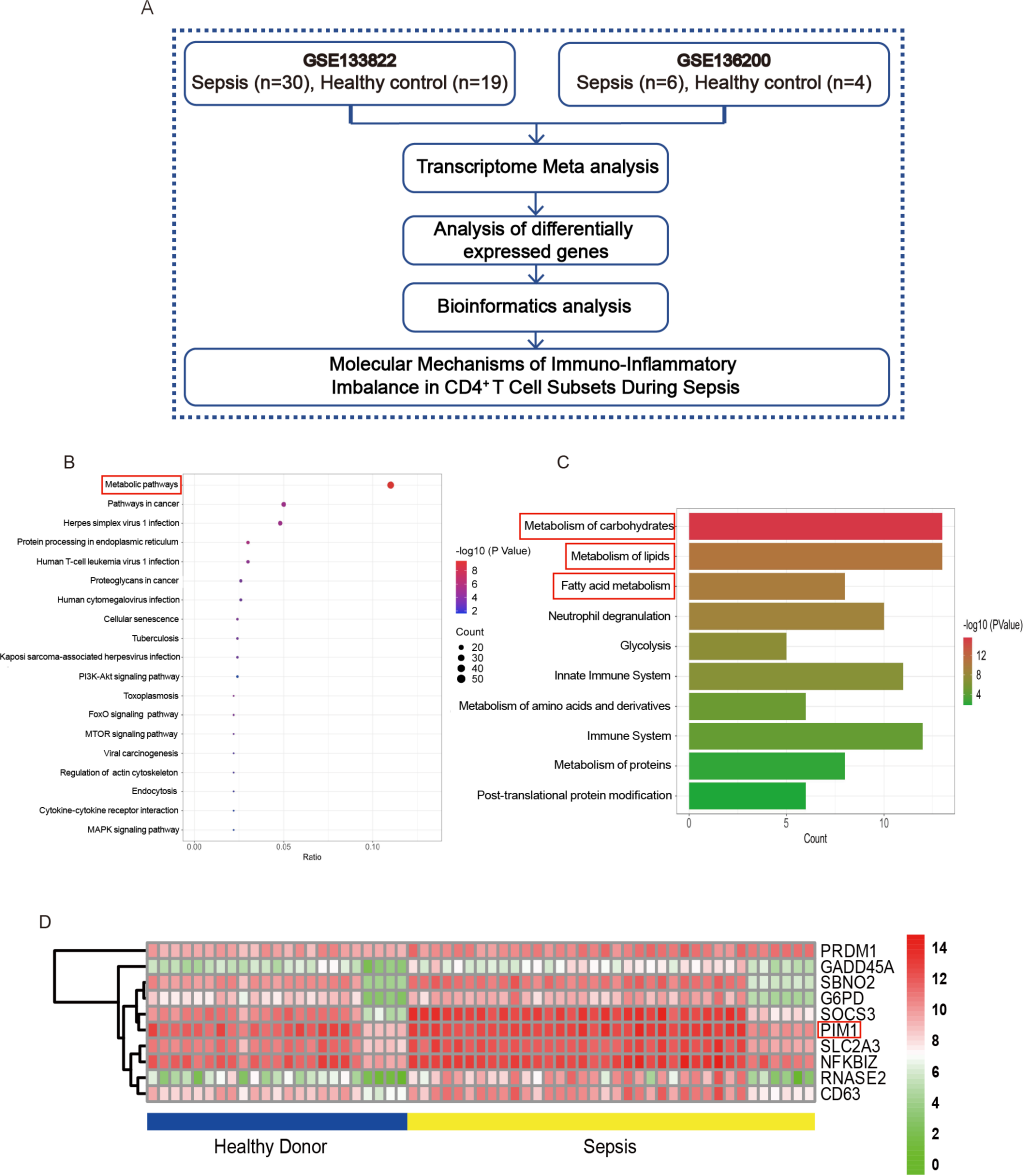
PIM1 expression is altered in sepsis-associated CD4^+^ T cells by transcriptome meta-analysis. (**A**) Workflow diagram of the transcriptome meta-analysis. (**B**) KEGG analysis of DEGs. (**C**) Metabolic pathway analysis of metabolism-related differential genes. (**D**) Heatmap of the top 10 DEGs.

To elucidate the molecular mechanisms underlying inflammatory imbalance within CD4^+^ T cell subpopulations during sepsis, we initially conducted a comprehensive transcriptome meta-analysis of CD4^+^ T cell data sets (GSE133822 and GSE136200) sourced from the National Center for Biotechnology Information Gene Expression Omnibus (NCBI GEO) database. This analysis encompassed samples derived from both sepsis patients and healthy donors, with the primary objective of identifying key differentially expressed genes (DEGs) associated with the inflammatory imbalance of CD4^+^ T cells (Fig. 1A). A total of 4,906 DEGs were identified between CD4^+^ T cells from sepsis patients and healthy controls (*P* < 0.05). Subsequently, we employed the KOBAS 3.0 online tool (http://bioinfo.org/kobas) to perform KEGG enrichment analysis on the top 500 DEGs to identify the significant pathways (Fig. 1A). KEGG analysis showed that the main enriched pathways were annotated as “metabolic pathways” of CD4^+^ T cells from sepsis compared with healthy controls (Fig. 1B).

To further identify the specific metabolic alterations in CD4^+^ T cells during sepsis, Reactome (https://reactome.org) was used to analyze the DEGs within metabolic pathways. Remarkably, our findings revealed significant alterations within the “metabolism of carbohydrates,” “metabolism of lipids,” and “fatty acid metabolism” in CD4^+^ T cells derived from sepsis patients (Fig. 1C). We next screen the DEGs, and the top 10 DEGs are shown in the heatmap (Fig. 1D). We found that the expression of PIM1 was significantly different between sepsis patients and healthy donors (*P* < 0.001). Studies have shown that PIM1 kinases can regulate various aspects of cellular metabolism in immune cells, including T cells and myeloid-derived suppressor cells (MDSCs) (27). The distinct expression levels of PIM1 observed between sepsis patients and healthy controls suggested the potential role of PIM1 in the pathogenesis of sepsis.

### PIM1 expression on CD4^+^ T cells is strongly correlated with the disease severity of sepsis

**Fig 2:**
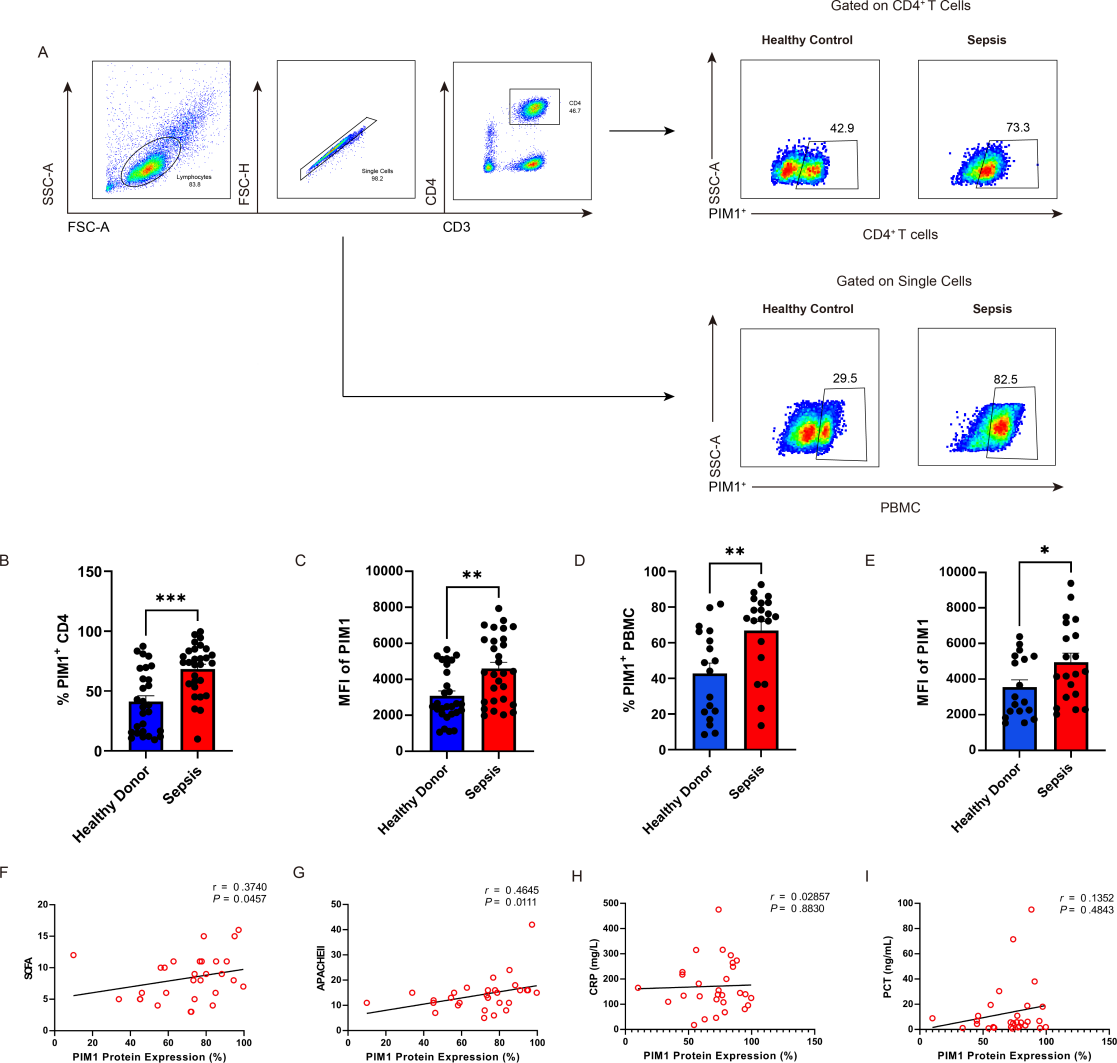
PIM1 expression is correlated with the disease severity of sepsis. (**A, B, and C**) Percentage (*P* = 0.0002) and MFI (*P* = 0.0022) of PIM1 protein expression on CD4^+^ T cells from sepsis patients and healthy donors. Healthy donor (*n* = 28) and sepsis (*n* = 29). (**D and E**) Percentage (*P* = 0.0021) and MFI (*P* = 0.0412) of PIM1 protein expression on PBMCs from sepsis patients and healthy donors. Healthy donor (*n* = 18) and sepsis (*n* = 20). (**F and G**) Correlation analysis between PIM1 protein expression and SOFA score (*n* = 29, *P* = 0.0457) and APACHE II score (*n* = 29, *P* = 0.0111). (**H and I**) Correlation analysis of PIM1 protein expression with plasma CRP (*n* = 29, *P* = 0.8830) and PCT concentrations (*n* = 29, *P* = 0.4843). Statistical significance was determined using the Mann-Whitney *U* test. Data are represented as mean ± SEM. *P < 0.05, **P < 0.01, ***P < 0.001.

PIM1 is a member of the serine/threonine kinase family and is involved in the control of cell growth, differentiation, and apoptosis (28). Previous studies showed PIM1 was involved in inflammatory bowel disease (IBD) and other inflammatory diseases (29), and we supposed that PIM1 is involved in the pathogenesis of sepsis. We first validated the expression of PIM1 on CD4^+^ T cells and PBMCs of sepsis patients. The expression of the PIM1 protein on CD4^+^ T cells was assessed using flow cytometry. Our study demonstrated that the proportion of PIM1-positive cells (Fig. 2A and B) and the mean fluorescence intensity (MFI) of PIM1 (Fig. 2C) were both considerably elevated in CD4^+^ T cells derived from sepsis patients in comparison to those from healthy donors, corroborating the findings from our transcriptome meta-analysis. Consistent results were observed in PBMCs (Fig. 2A, D and E) and CD8^+^ (CD3^+^CD4^-^) T cells (Fig. S1A through C).

To further elucidate the relationship between PIM1 expression levels and the severity of sepsis, we conducted a correlation analysis between PIM1 and the clinical parameters indicating severity and prognosis, including APACHE II, SOFA score, and levels of plasma C-reactive protein (CRP) and procalcitonin (PCT) concentration. Interestingly, our results revealed a significant positive correlation between PIM1 expression and SOFA score (*P* = 0.0457, *r* = 0.3740) (Fig. 2F) and APACHE II score (*P* = 0.0111, *r* = 0.4645) (Fig. 2G), indicating that PIM1 can effectively regulate disease progression in sepsis. Nevertheless, no significant correlation was detected between PIM1 expression in CD4^+^ T cells and the plasma levels of CRP (Fig. 2H) and PCT (Fig. 2I). These results demonstrate that PIM1 expression is elevated on CD4^+^ T cells in sepsis patients and correlates with the progression of the disease, suggesting the crucial role of PIM1 in the progression of sepsis.

### PIM1 promotes the imbalance of CD4^+^ T cell subsets in sepsis

**Fig 3:**
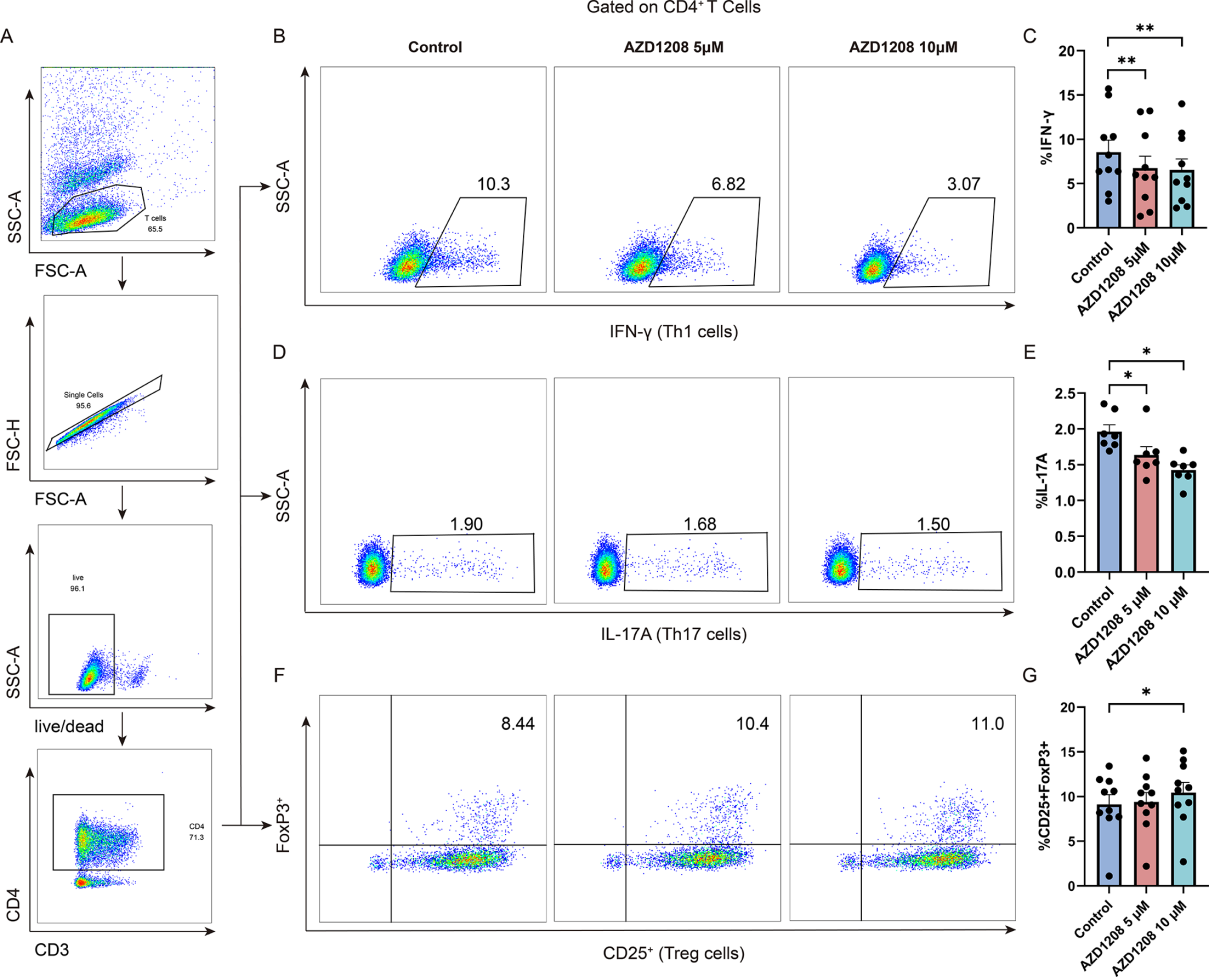
PIM1 is involved in regulating Th1, Th17, and Treg subsets imbalance. (**A**) The gating strategy for CD4^+^ T cells. (**B and C**) The change of CD4^+^IFN-γ^+^ Th1 cells expression level after inhibiting PIM1 kinase activity (*n* = 10, Control vs AZD1208 5 µM, *P* = 0.0039; Control vs AZD1208 10 µM, *P* = 0.0020). (**D and E**) Changes in the expression level of CD4^+^IL-17A^+^ Th17 cells after inhibiting PIM1 kinase activity (*n* = 7, Control vs AZD1208 5 µM, *P* = 0.0156; Control vs AZD1208 10 µM, *P* = 0.0313). (**F and G**) Changes in expression level of CD4^+^CD25^+^FoxP3^+^ Treg cells after inhibiting of PIM1 kinase activity (*n* = 10, Control vs AZD1208 5 µM, *P* = 0.5566; Control vs AZD1208 10 µM, *P* = 0.0137). *P* value was calculated by the Wilcoxon signed-rank test. Data are plotted as mean ± SEM. *P < 0.05, **P < 0.01.

To further elucidate the role of PIM1 in sepsis, investigate the effect of PIM1 on the differentiation of Th1, Th17, and Treg cells, as well as the dysregulation of Th1, Th17, and Treg subsets, and different concentrations (5 µM, 10 µM) of the chemical inhibitor of PIM1 kinase, AZD1208, were added to the activated CD3^+^ T cells to inhibit the kinase activity of PIM1 after determining the IC50 by CCK8 experiment (Fig. S2). Subsequently, we assessed the effects of PIM1 on Th1, Th17, and Treg differentiation after 24 h of incubation. The portions of IFN-γ^+^ Th1 (Fig. 3A through C) and IL-17A^+^ Th17 (Fig. 3D and E) cells were decreased significantly after inhibition of PIM1 kinase activity, indicating a decreased differentiation of inflammatory IFN-γ^+^ Th1 and IL-17A^+^ Th17 cells. In contrast, the proportion of the CD25^+^FoxP3^+^ Treg subset increased (Fig. 3F and G). To further verify the regulatory effect of PIM1 on Th1, Th17, and Treg cells, we knocked down PIM1 using siRNA transfection and examined the expression of IFN-γ^+^ Th1, IL-17A^+^ Th17 cells, and CD25^+^FoxP3^+^ Treg cells. Our observations indicated a reduction in the proportion of IFN-γ-producing Th1 (Fig. S3A through C) and IL-17A-producing Th17 cells (Fig. S3D and E), whereas there was a corresponding increase in CD25^+^FoxP3^+^ Treg cells (Fig. S3F and G). In addition, we analyzed the inflammatory, exhausted states, and cytotoxic ability of CD4^+^ T cells in sepsis patients by detecting the IL-22, PD-1, TIM-3, and Granzyme B expression. We found that the expressions of IL-22, PD-1, and TIM-3 in CD4^+^ T cells of sepsis patients were significantly higher than that of healthy controls (Fig. S4A through F), suggesting that CD4^+^ T cells in sepsis patients are in a hyperinflammatory state and more prone to exhaustion. Granzyme B expression in CD4^+^ T cells showed an increasing trend in sepsis patients, but the difference was not statistically significant (Fig. S4G and H). CD4^+^ T cells derived from sepsis patients exhibited diminished Ki-67 expression and reduced secretion of IFN-γ, indicative of impaired functional capacity upon *in vitro* stimulation (Fig. S4I through L). These results provide evidence that PIM1 plays a crucial role in regulating the imbalance of Th1, Th17, and Treg cells during sepsis, thus highlighting its potential role in regulating the inflammatory state and immune balance in patients with sepsis.

### ABCG1 is involved in Th1, Th17, and Treg imbalance induced by PIM1

**Fig 4:**
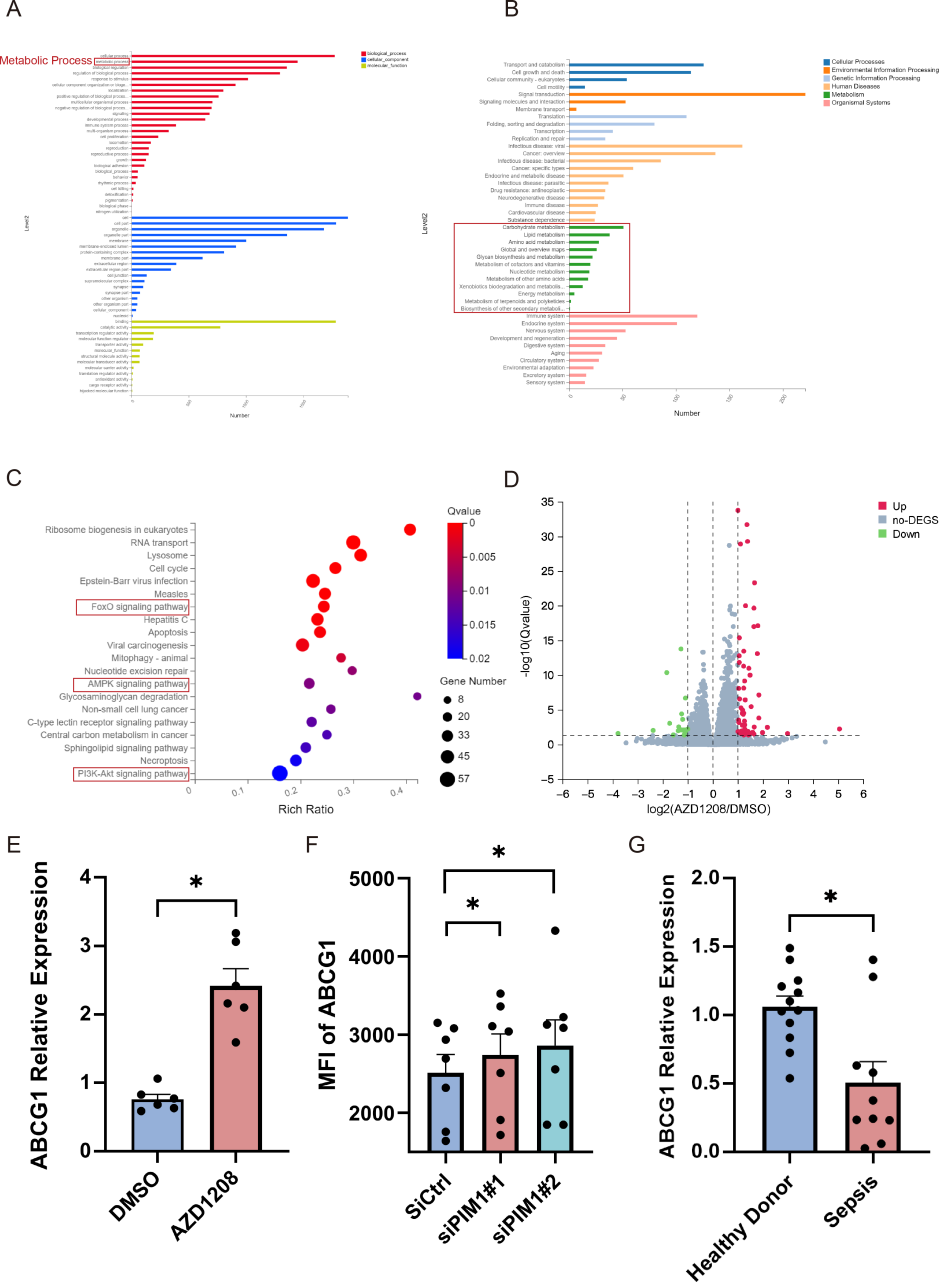
Bulk RNA sequencing analysis of CD4^+^ T cells after inhibition of PIM1 kinase activity. (**A and B**) KEGG analysis of DEGs. (**C**) GO analysis of DEGs. (**D**) Volcanic maps of DEGs. (**E**) ABCG1 mRNA expression in CD4^+^ T cells after inhibiting PIM1 kinase activity (*n* = 6, *P* = 0.0313). (**F**) MFI of ABCG1 protein expression on CD4^+^ T cells after PIM1 siRNA transfection (*n* = 7, siCtrl vs siPIM1#1, *P =* 0.0313; siCtrl and siPIM1#2, *P =* 0.0313). (**G**) ABCG1 mRNA expression (*P* value = 0.0112) of CD4^+^ T cells in sepsis and healthy donors. Healthy Donor (*n* = 12) and sepsis (*n* = 10). For panels **E** and **F**, *P* value was calculated by the Wilcoxon signed rank test. For panel **G**, *P* value was calculated by Mann-Whitney *U* test. Data are plotted as mean ± SEM. *P < 0.05.

To gain a more in-depth understanding of how PIM1 regulates Th1, Th17, and Treg imbalance, we further analyze the different genes and the pathways of CD4^+^ T cells after inhibiting PIM1 by bulk RNA-sequencing analysis. CD4^+^ T cells isolated from healthy donors were activated with anti-CD3/CD28 beads for 24 h in the presence of AZD1208, a PIM1 kinase activity inhibitor. The cells were then harvested for RNA-sequencing analysis. The KEGG pathway enrichment analysis revealed that the altered expressed genes significantly enriched the metabolic pathways, especially in the lipid metabolism pathway (Fig. 4A and B). GO analysis revealed that some of the crux metabolic regulatory pathways, including the “Foxo signaling pathway,” “AMPK signaling pathway,” and “PI3K-Akt signaling pathway,” changed significantly (Fig. 4C). Upon further analysis of the genes that revealed significant changes (fold change > 2, *Q* value < 0.05), a total of 55 genes were significantly upregulated, and 20 genes were significantly downregulated (Fig. 4D; Fig. S5). By comparing the gene expression profiles between the AZD1208-treated group and the control group, we identified a significant upregulation of the cholesterol metabolism gene, ATP-binding cassette transporter G1 (ABCG1), following PIM1 inhibition (Fig. 4E). This finding suggests that PIM1 may regulate ABCG1 and thus potentially influence the cholesterol metabolic processes within CD4^+^ T cells. To further validate the regulatory role of PIM1 on ABCG1, we performed siRNA-mediated PIM1 knockdown experiments and subsequently examined the ABCG1 expression in CD4^+^ T cells. The results demonstrated that PIM1 knockdown led to an increase in ABCG1 expression (Fig. 4F).

Since we found that PIM1 was elevated in CD4^+^ T cells during sepsis, we speculated that PIM1 may cause the decreased expression of ABCG1 in CD4^+^ T cells of sepsis patients. Then, we analyzed the ABCG1 mRNA expression in CD4^+^ T cells from septic patients and healthy controls. We found that ABCG1 was significantly decreased in septic patients compared with healthy donors (Fig. 4G). In addition, to further examine the cholesterol metabolism of CD4^+^ T cells in sepsis, the key genes involved in cholesterol metabolism were examined, including liver X receptor β (LXR β), sterol response element-binding protein (SREBP), SREBP cleavage-activating protein (SCAP), and acetyl-coenzyme A acetyltransferases 1 (ACAT1). We observed that except for ACAT1 mRNA, which exhibited a slight decrease, the mRNA levels of all other cholesterol-related genes were significantly reduced in CD4^+^ T cells derived from sepsis patients compared with those from healthy controls (Fig. S6A through D). These findings suggest that aberrant cholesterol metabolism in CD4^+^ T cells of sepsis patients and cholesterol metabolism may be involved in regulating the balance of CD4^+^ T subsets.

**Fig 5:**
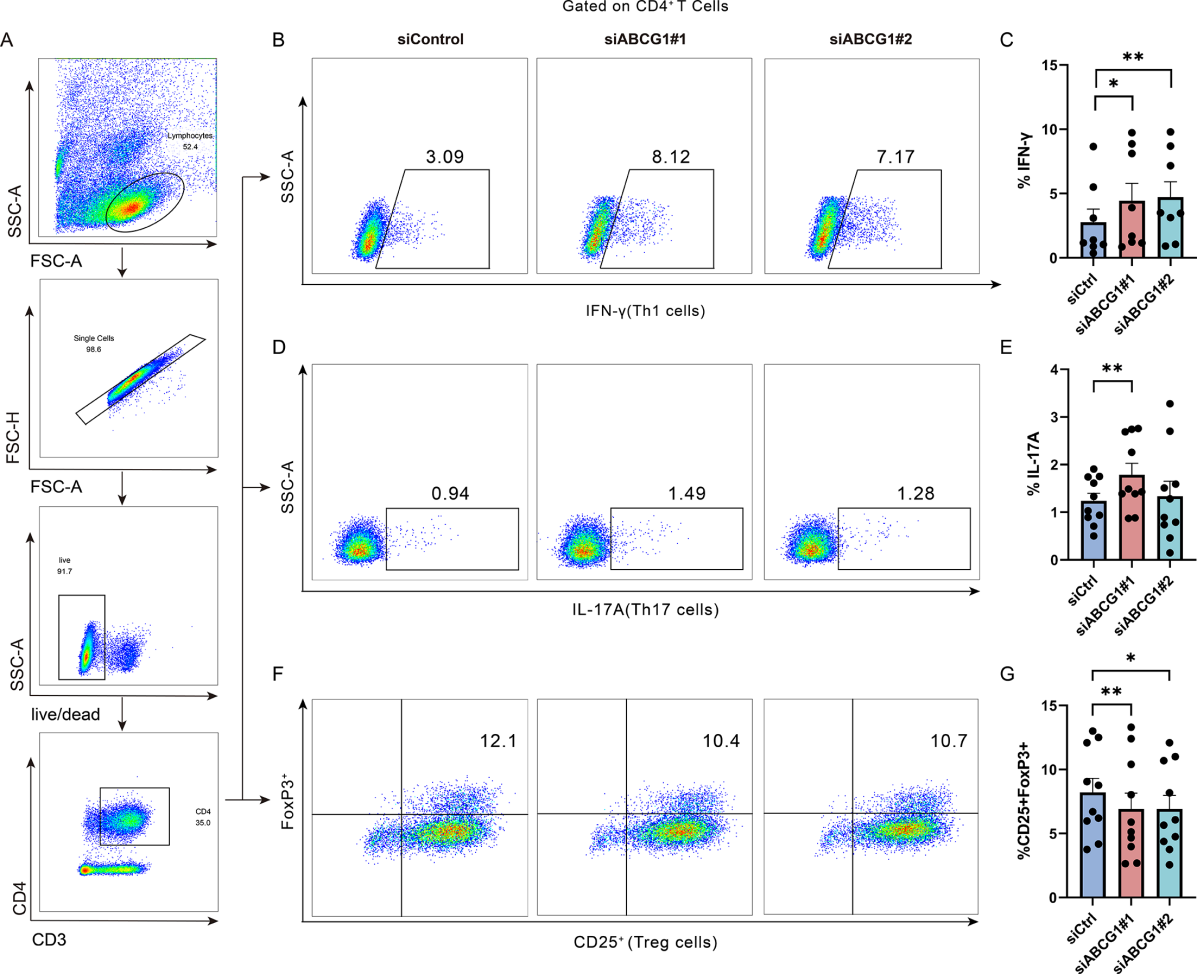
ABCG1 modulates the imbalance in Th1, Th17, and Treg cell subsets. (**A**) The gating strategy for CD4^+^ T cells following ABCG1 siRNA transfection via electroporation. (**B and C**) Changes in expression level of CD4^+^IFN-γ^+^ Th1 cells after knocking down ABCG1 (*n* = 8, siCtrl vs siABCG1#1, *P* = 0.0234; siCtrl vs siABCG1#2, *P* = 0.0078). (**D and E**) Changes in expression level of CD4^+^IL-17A^+^ Th17 cells expression level after knocking down ABCG1 (*n* = 10, siCtrl vs siABCG1#1, *P* = 0.0098; siCtrl vs siABCG1#2, *P* = 0.7695). (**F and G**) Changes in expression level of CD4^+^CD25^+^FoxP3^+^ Treg cells after knocking down ABCG1 (*n* = 10, siCtrl vs siABCG1#1, *P* = 0.0098; siCtrl vs siABCG1#2, *P* = 0.0137). *P* value was calculated by the Wilcoxon signed-rank test. Data are plotted as mean ± SEM. *P < 0.05, **P < 0.01.

To further demonstrate the role of ABCG1 in mediating PIM1’s regulatory effects on Th1, Th17, and Treg cells, we utilized siRNA transfection to knock down ABCG1 and subsequently assessed the effects on IFN-γ^+^ Th1 cells, IL-17A^+^ Th17 cells, and CD25^+^FoxP3^+^ Treg cells. The results revealed that ABCG1 knockdown led to an increase in the proportions of IFN-γ^+^ Th1 (Fig. 5A through C) and IL-17A^+^ Th17 cells (Fig. 5D and E), whereas the proportion of CD25^+^FoxP3^+^ Treg cells decreased (Fig. 5F and G). These findings are contrary to the effects observed upon PIM1 kinase inhibition and PIM1 knockdown, indicating that ABCG1-mediated cholesterol metabolism is implicated in the PIM-induced imbalance of Th1, Th17, and Treg cells.

### PIM1 is involved in regulating cholesterol metabolism in CD4^+^ T cells

**Fig 6:**
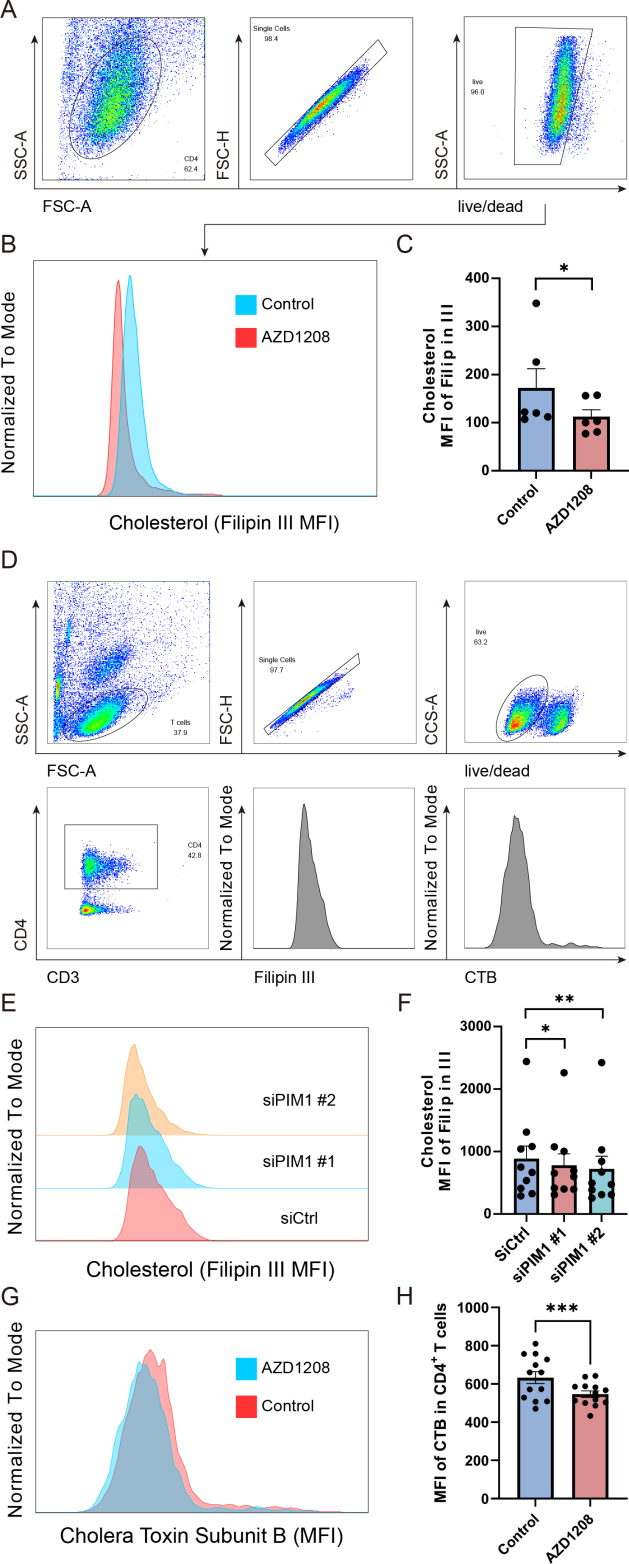
PIM1 regulates cholesterol metabolism in CD4^+^ T cells. (**A**) Gating strategy for filipin III expression in CD4^+^ T cells. (**B and C**) Intracellular cholesterol staining with filipin III in CD4^+^ T cells following inhibition of PIM1 kinase activity (*n* = 6, *P* = 0.0313). (**D**) Gating strategy for filipin III and cholera toxin subunit B (CTB) expression in CD4^+^ T cells following PIM1 siRNA transfection or PIM1 kinase inhibition in CD3^+^ T cells. (**E and F**) Intracellular cholesterol staining with filipin III in CD4^+^ T cells after PIM1 siRNA transfection (*n* = 10, siCtrl vs siPIM1#1, *P* = 0.0371; siCtrl vs siPIM1#2, *P* = 0.0059). (**G and H**) Lipid rafts detection by AF488-conjugated CTB on CD4^+^ T cells following PIM1 kinase inhibition (*n* = 13, *P* = 0.0010). Statistical significance was determined using the Wilcoxon signed-rank test. Data are represented as mean ± SEM. *P < 0.05, **P < 0.01, ***P < 0.001.

Since our previous results suggested that PIM1 probably induces Th1, Th17, and Treg imbalance by regulating the cholesterol metabolism in CD4^+^ T cells, to gain further insight into whether PIM1 regulates the cholesterol metabolism levels in CD4^+^ T cells, we detected the intracellular cholesterol content after inhibiting PIM1 kinase activity of CD4^+^ T cells. Intracellular cholesterol levels were detected using filipin III staining and subsequently quantified via flow cytometry analysis. The results revealed that the MFI of filipin III in CD4^+^ T cells significantly decreased after the inhibition of PIM1 kinase activity (Fig. 6A through C), that is to say, the intracellular cholesterol content was decreased in CD4^+^ T cells. From the images obtained by imaging flow cytometry, it can be more clearly seen that the intracellular cholesterol in CD4^+^ T cells was significantly reduced after the inhibition of PIM1 kinase activity (Fig. S7). In addition, we further examined the changes in intracellular cholesterol in CD4^+^ T cells after knocking down PIM1 by siRNA transfection with two specifically designed siRNA pairs targeting different regions of the PIM1 gene. Similar results were observed; the MFI value of FlipinIII in CD4^+^ T cells was significantly decreased, which was in line with the results of inhibition of PIM1 kinase activity (Fig. 6D through F). Given that cholesterol is an important component of lipid rafts, we further employed a fluorescently conjugated Cholera Toxin Subunit B (CTB) molecule to detect lipid rafts. PIM1 inhibition resulted in a significant decrease in CTB fluorescence intensity, revealing a reduction in lipid raft abundance on the surface of CD4^+^ T cells (Fig. 6D, G and H). These findings suggest that both the downregulation of PIM1 expression and the inhibition of its kinase activity effectively reduce intracellular cholesterol levels in CD4^+^ T cells, highlighting the significant role of PIM1 in regulating cholesterol metabolism in CD4^+^ T cells.

### The Th1, Th17, and Treg imbalance induced by PIM1 is in a cholesterol-dependent manner

**Fig 7:**
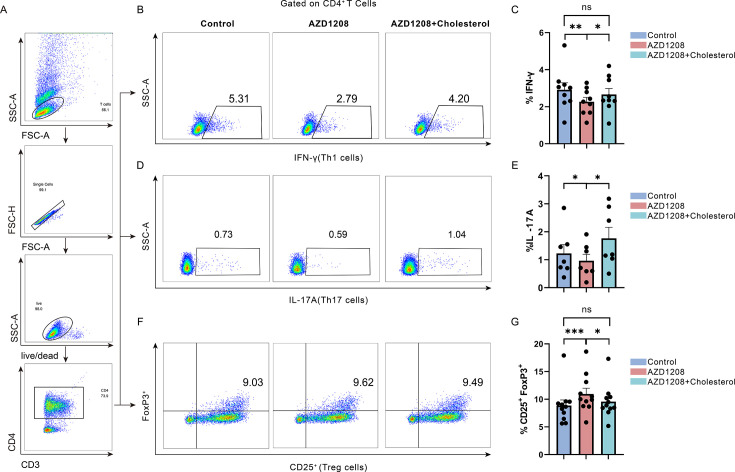
PIM1 induces the inflammatory imbalance in CD4^+^ T cells via cholesterol-dependent mechanisms. (**A**) The gating strategy for CD4^+^ T cells. (**B and C**) Expression of CD4^+^IFN-γ^+^ Th1 cells following PIM1 kinase inhibition and cholesterol supplementation (*n* = 9, Control vs AZD1208, *P* = 0.0039; AZD1208 vs AZD1208+ Cholesterol, *P* = 0.0273). (**D and E**) Expression of CD4^+^IL-17A^+^ Th17 cells following PIM1 kinase inhibition and cholesterol supplementation (*n* = 7, Control vs AZD1208, *P* = 0.0156; AZD1208 vs AZD1208+ Cholesterol, *P* = 0.0156). (**F and G**) Expression of CD4^+^CD25^+^FoxP3^+^ Treg cells following PIM1 kinase inhibition and cholesterol supplementation (*n* = 11, Control vs AZD1208, *P* = 0.0010; AZD1208 vs AZD1208+ Cholesterol, *P* = 0.0186). Statistical significance was assessed using the Wilcoxon signed-rank test. Data are presented as mean ± SEM. *P < 0.05, **P < 0.01, ***P < 0.001, ns: not significant.

Recent research has highlighted the emerging role for cholesterol as an important modulator in both innate and adaptive immune responses (21). In addition to its role in facilitating immune synapse formation and cellular activation via cell membrane lipid rafts, research has shown that the cholesterol pathway plays a significant part in regulating the differentiation of CD4^+^ T cell subsets (30). We next determined whether PIM1 regulates the Th1, Th17, and Treg imbalance through the regulation of cholesterol metabolism. To examine whether the addition of exogenous cholesterol rescued the Th1, Th17, and Treg imbalance after PIM1 inhibition, CD4^+^ T cells were activated in the presence of 50 µg/mL exogenous cholesterol after inhibiting the PIM1 kinase activity with AZD1208. As shown in Fig. 7, when only inhibiting the PIM1 kinase, the portion of CD4^+^IFN-γ^+^ Th1 and CD4^+^IL-17A^+^ Th17 cells decreased significantly, and the expression of CD4^+^CD25^+^FoxP3^+^ was increased. Interestingly, we found that the portion of CD4^+^IFN-γ^+^ Th1 (Fig. 7A through C) and CD4^+^IL-17A^+^ Th17 (Fig. 7D and E) cells was recovered, and the CD4^+^CD25^+^FoxP3^+^ (Fig. 7F and G) decreased after cholesterol supplementation to CD4^+^ T cells. Taken together, these findings suggested that PIM1 regulates Th1, Th17, and Treg imbalance through the regulation of cholesterol metabolism.

## DISCUSSION

As a disease with extremely high mortality and scarce efficient treatment, sepsis remains a significant global health burden (2). The imbalanced CD4^+^ T cell subsets contribute to uncontrolled inflammation, which usually results in multiple organ dysfunctions and death of patients with sepsis. In this study, we first identified PIM1 as a key molecule in regulating CD4^+^ T cell inflammatory imbalance in sepsis. We have demonstrated that expression levels of PIM1 are significantly elevated in CD4^+^ T cells from sepsis patients and exhibit a strong correlation with the severity of the disease. Additionally, the proportions of Th1 and Th17 cells were observed to decrease following PIM1 inhibition or knockdown, whereas the proportion of Treg cells was found to increase. Our research further indicates that upregulation of PIM1 may increase intracellular cholesterol content in CD4^+^ T cells, potentially through reducing cholesterol efflux mediated by the ABCG1 transporter. Moreover, cholesterol supplementation effectively reversed the regulatory impact of PIM1 on the balance of Th1, Th17, and Treg cell subsets.

We first identified PIM1 as a significantly upregulated gene in CD4^+^ T cells from sepsis patients by conducting a comprehensive transcriptome meta-analysis. PIM1, a member of the PIM kinase family, is a constitutively active serine/threonine kinase that plays a pivotal role in regulating cell proliferation, apoptosis, and migration (31). PIM1 is overexpressed in a variety of cancer cells and is associated with the development and progression of several hematological malignancies and solid tumors (32). PIM kinases are considered a downstream effector of many cytokine signaling pathways, including the JAK-STAT pathway (33). Previous studies have documented that PIM1 has been reported to participate in infectious diseases and autoimmune diseases in animal models by regulating CD4^+^ T cell subsets (34, 35). However, the conclusions were controversial. Teija et al. (36) showed that the cytokines promoting Th1 can selectively upregulate the expression of human pim-family genes. Wang et al. (37) showed that *in vitro* inhibition of PIM1 kinase prevented intestinal inflammation and attenuated Th17 differentiation and cytokine production, whereas the Th1 subsets were unaffected. In sepsis, a severe inflammatory condition, the specific alterations of PIM1, along with its regulatory functions and the underlying mechanisms influencing disease progression, have not been previously reported. In this study, we have shown that inhibiting PIM1 kinase activity or reducing PIM1 expression leads to a decrease in the differentiation of Th1 and Th17 cells while enhancing the differentiation of Treg cells in human CD4^+^ T cells. Consequently, the elevated expression of PIM1 observed in CD4^+^ T cells from sepsis patients may result in increased production of proinflammatory cytokines and activation of cytokine signaling pathways, contributing to the inflammatory response in sepsis.

Next, our study unveiled the involvement of PIM1 in cholesterol metabolism within CD4^+^ T cells. Through bulk RNA sequencing analysis, we discovered that the expression of ABCG1 on CD4^+^ T cells was significantly increased following the inhibition of PIM1 kinase activity or knocking down PIM1 and was notably reduced in the context of sepsis. ABCG1 is a member of the ABC transporter family, which plays a crucial role in regulating cellular cholesterol homeostasis by promoting cholesterol efflux (38, 39). We further revealed that inhibiting PIM1 or knocking down PIM1 caused the intracellular cholesterol content to decrease significantly in CD4^+^ T cells. Cholesterol and its various metabolites exert complex biological functions in immune cells, and previous studies have shown the dysregulated cholesterol metabolism in sepsis (40, 41). Although the role of PIM1 in governing cholesterol metabolism during sepsis has not been elucidated, previous studies have revealed that PIM1 orchestrates a diverse array of metabolic pathways, including the activation of mTORC, inhibition of AMPK, and regulation of PPARγ expression (27, 42–44). These pathways are likely to be further implicated in stabilizing LXR and activating SREBP, thereby playing a crucial role in regulating cholesterol homeostasis (42, 45). LXR is a key regulator of cholesterol efflux, primarily through the upregulation of ABCG1 expression. Our investigation has uncovered a significant reduction in LXRβ mRNA levels in CD4^+^ T cells from sepsis patients, which aligns with the observed alterations in ABCG1 expression. These results indicate that PIM1 may influence the expression of ABCG1, at least in part, by modulating the LXR signaling pathway. Collectively, these findings suggest that PIM1 may serve as a critical metabolic regulator in orchestrating cholesterol metabolism under septic conditions.

Furthermore, we demonstrated that PIM1 might regulate Th1, Th17, and Treg imbalance through ABCG1-mediated cholesterol metabolism in sepsis. Previous studies have demonstrated the crucial role of cholesterol and cholesterol derivatives in modulating the formation of the immunological synapse and T-cell activation in addition to maintaining cell membrane stiffness (46, 47); moreover, the increases in intracellular cholesterol favored inflammatory differentiation of CD4^+^ T cells (22). However, the role of cholesterol metabolism in the differentiation of Treg cells has so far been inconclusive. Surls et al. (48) observed that the elevation of membrane cholesterol favored the differentiation of Th1 cells from CD4 T-cells without impacting the suppressive function of Foxp3^+^ regulatory T-cells. Herold et al. (49) reported that the activation of LXR in T cells, leading to a decrease in intracellular cholesterol levels, effectively suppressed the polarization of Th1 and Th17 cells *in vitro*, but significantly induced Treg cell differentiation in a receptor-specific manner. In contrast, Cheng et al. (50) observed that T cell-specific deficiency in ABCG1 resulted in an elevated percentage of Treg cells within the aorta and aorta-draining lymph nodes, consequently leading to reduced atherosclerosis in Ldlr^–/–^ mice. Wen et al. (51) demonstrated that nicotine notably increased ABCG1 expression and concurrently lowered intracellular cholesterol levels, which was related to a decrease in the frequency of Treg cells. Our study further revealed that inhibition of PIM1 leads to a reduction in intracellular cholesterol, which in turn causes a decrease in Th1 and Th17 differentiation while promoting the differentiation of Treg cells. The replenishment of cholesterol can reverse these changes in CD4^+^ T cell subsets, illustrating that PIM1 regulates the balance of CD4^+^ T cell subsets and inflammatory responses in a cholesterol-dependent manner. That is to say, the increased PIM1 expression on CD4^+^ T cells in sepsis promotes the inflammatory Th1 and Th17 cells and inhibits the Tregs differentiation by increasing the intracellular cholesterol content, thus exacerbating the pro-inflammatory and anti-inflammatory imbalance in sepsis.

In conclusion, this study reveals that PIM1 participated in regulating the imbalance of Th1, Th17, and Treg subsets, further promoting proinflammatory cytokine production and inflammatory and anti-inflammatory imbalance in sepsis. Additionally, our research indicated that the cholesterol metabolism, potentially mediated by ABCG1, is implicated in PIM1’s regulatory effect on the Th1, Th17, and Treg imbalance. The interplay between PIM1, cholesterol homeostasis, and CD4^+^ T cell subsets offers novel insights into the inflammatory imbalance during sepsis. Future research should delve deeper into the molecular mechanisms underlying the interaction between PIM1 and cholesterol metabolism. Although PIM1 inhibition has garnered significant interest due to its potential therapeutic applications in various cancers, particularly in hematological malignancies and solid tumors, the clinical application of inhibitors like AZD1208 is currently limited by significant adverse effects and challenges in achieving therapeutic efficacy as monotherapy (52, 53). Developing therapeutic strategies that combine PIM1 inhibition with cholesterol modulation may help regulate the immune-inflammatory cascade in sepsis, offering potential clinical benefits for patient management.

## SUPPLEMENTAL MATERIAL

Figure S1 mbio.01680-25-s0001.tif PIM1 expression on CD8^+^ T cells.

Figure S2 mbio.01680-25-s0002.tif Viability of CD3^+^ T cells treated with different concentrations of AZD1208.

Figure S3 mbio.01680-25-s0003.tif PIM1 is involved in regulating Th1, Th17, and Treg subset imbalance.

Figure S4 mbio.01680-25-s0004.tif The functional status of CD4^+^ T cell subsets in sepsis.

Figure S5 mbio.01680-25-s0005.tif DEGs of CD4^+^ T cells after inhibiting PIM1 kinase activity by RNA sequencing.

Figure S6 mbio.01680-25-s0006.tif mRNA expression of cholesterol metabolism-related genes in CD4^+^ T cells from sepsis patients.

Figure S7 mbio.01680-25-s0007.tif Cholesterol level in CD4^+^ T cells after inhibiting PIM1 kinase activity.

Legends mbio.01680-25-s0008.docx Supplemental figure legends.

Supplemental Tables mbio.01680-25-s0009.docx Tables S1 and S2.

ASM does not own the copyrights to Supplemental Material that may be linked to, or accessed through, an article. The authors have granted ASM a non-exclusive, world-wide license to publish the Supplemental Material files. Please contact the corresponding author directly for reuse.
