# Supplementary figures and images for: PIM1 orchestrates sepsis-associated inflammatory imbalance in CD4+ T cell subsets via cholesterol metabolism
Source: mBio. 2025 Sep 3;16(10):e01680-25. doi: 10.1128/mbio.01680-25 (PMC12505896; doi:10.1128/mbio.01680-25)

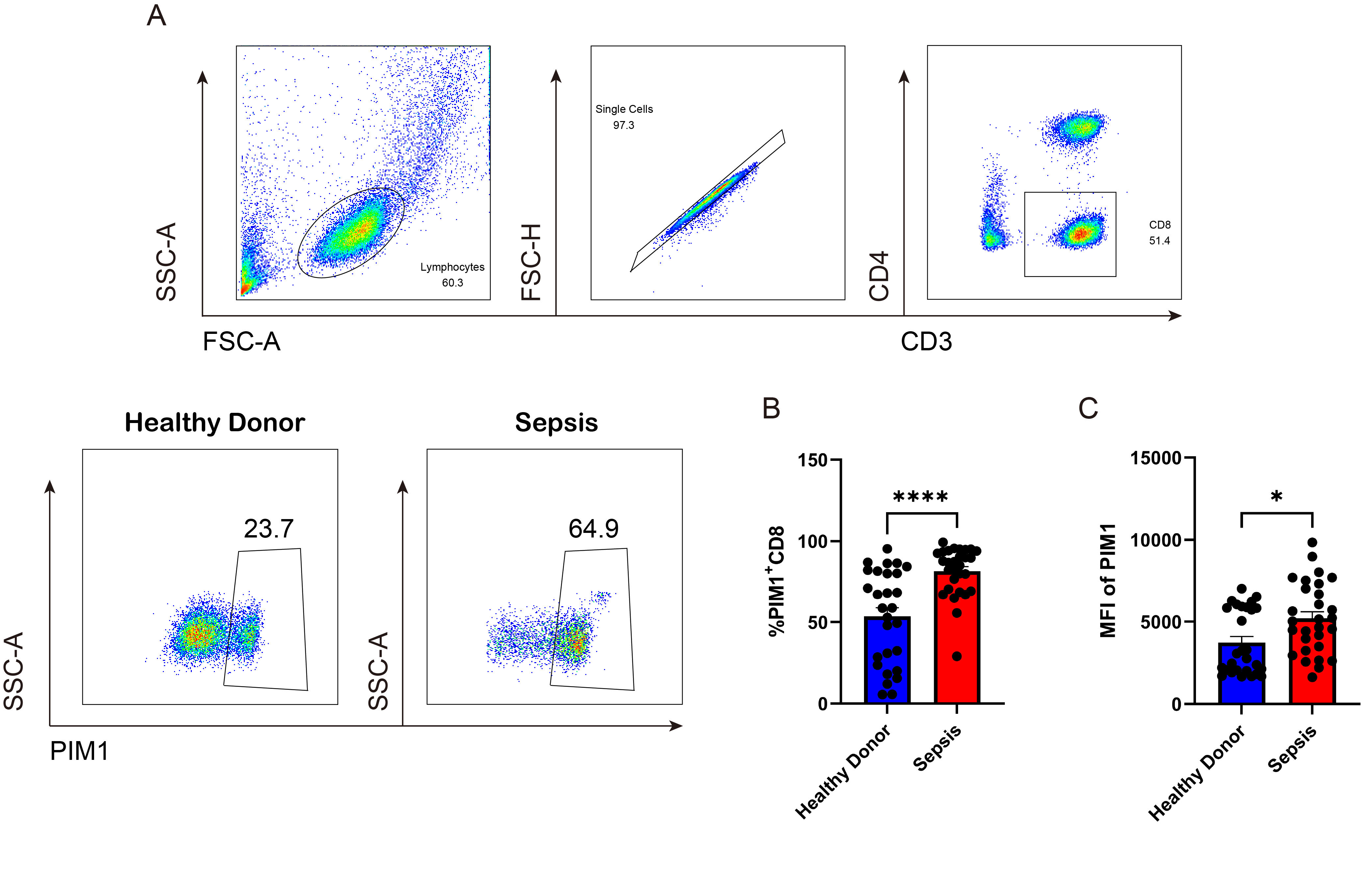

Supplement: Figure S1 — PIM1 expression on CD8+ T cells. [file mbio.01680-25-s0001.tif]

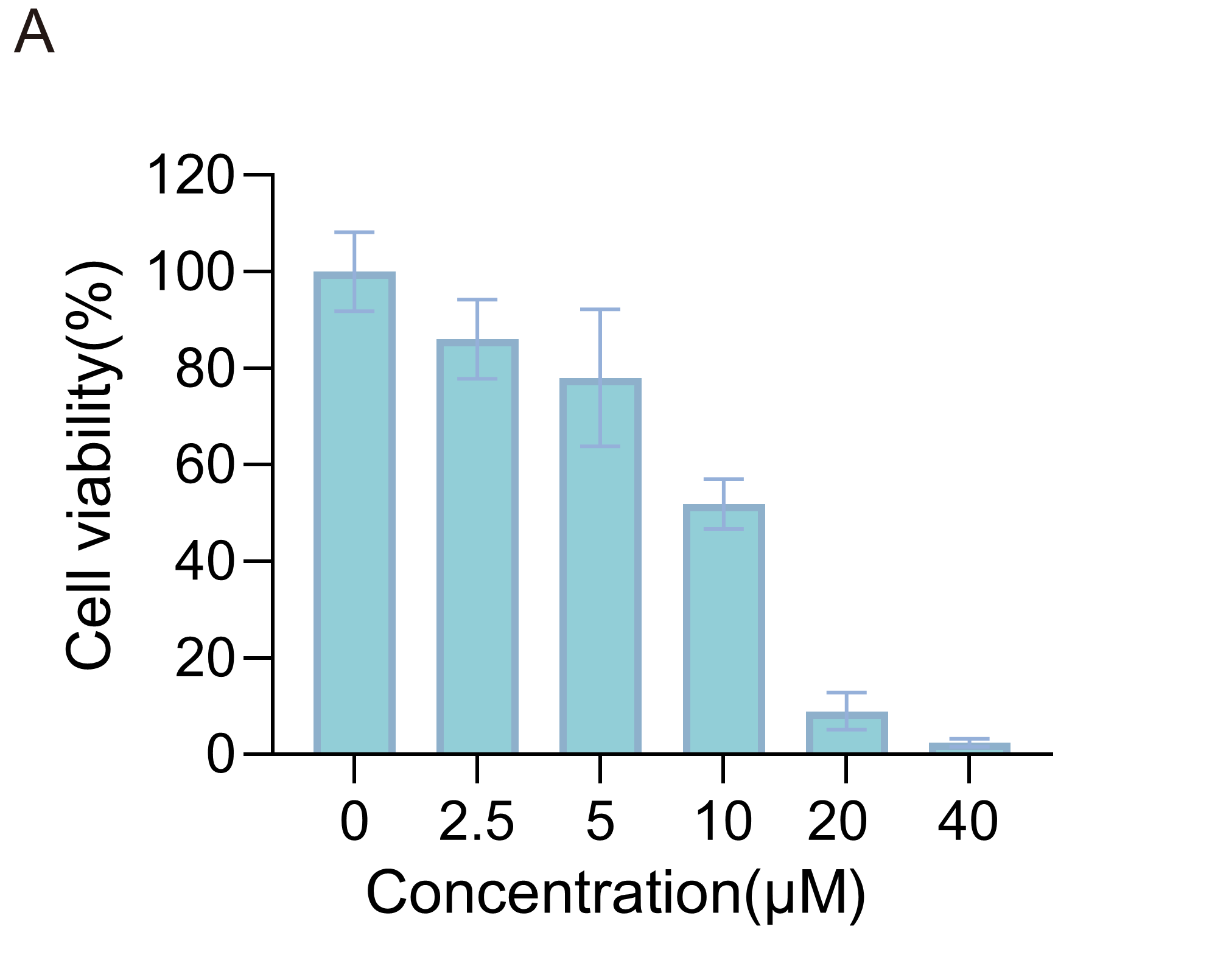

Supplement: Figure S2 — Viability of CD3+ T cells treated with different concentrations of AZD1208. [file mbio.01680-25-s0002.tif]

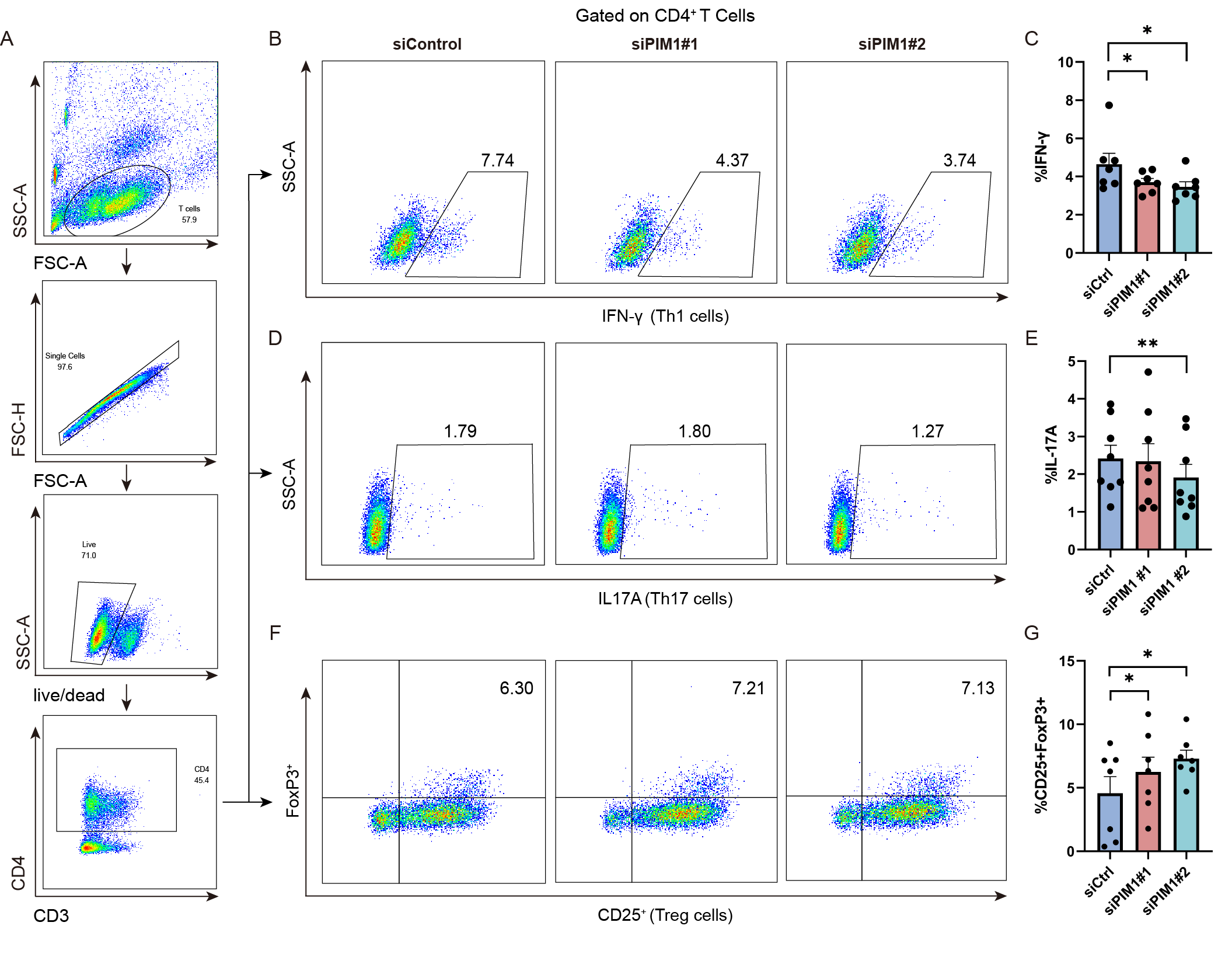

Supplement: Figure S3 — PIM1 is involved in regulating Th1, Th17, and Treg subset imbalance. [file mbio.01680-25-s0003.tif]

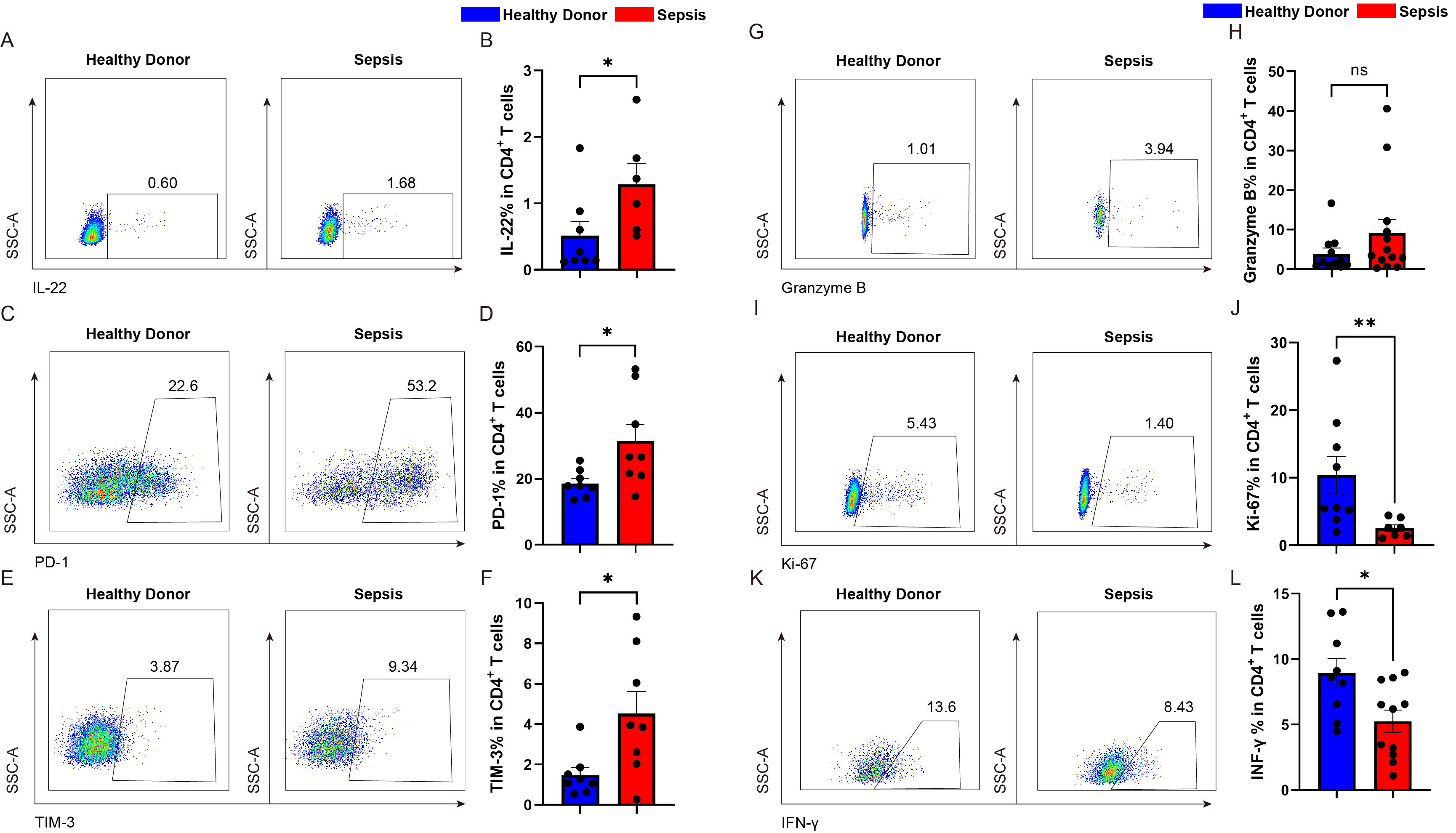

Supplement: Figure S4 — The functional status of CD4+ T cell subsets in sepsis. [file mbio.01680-25-s0004.tif]

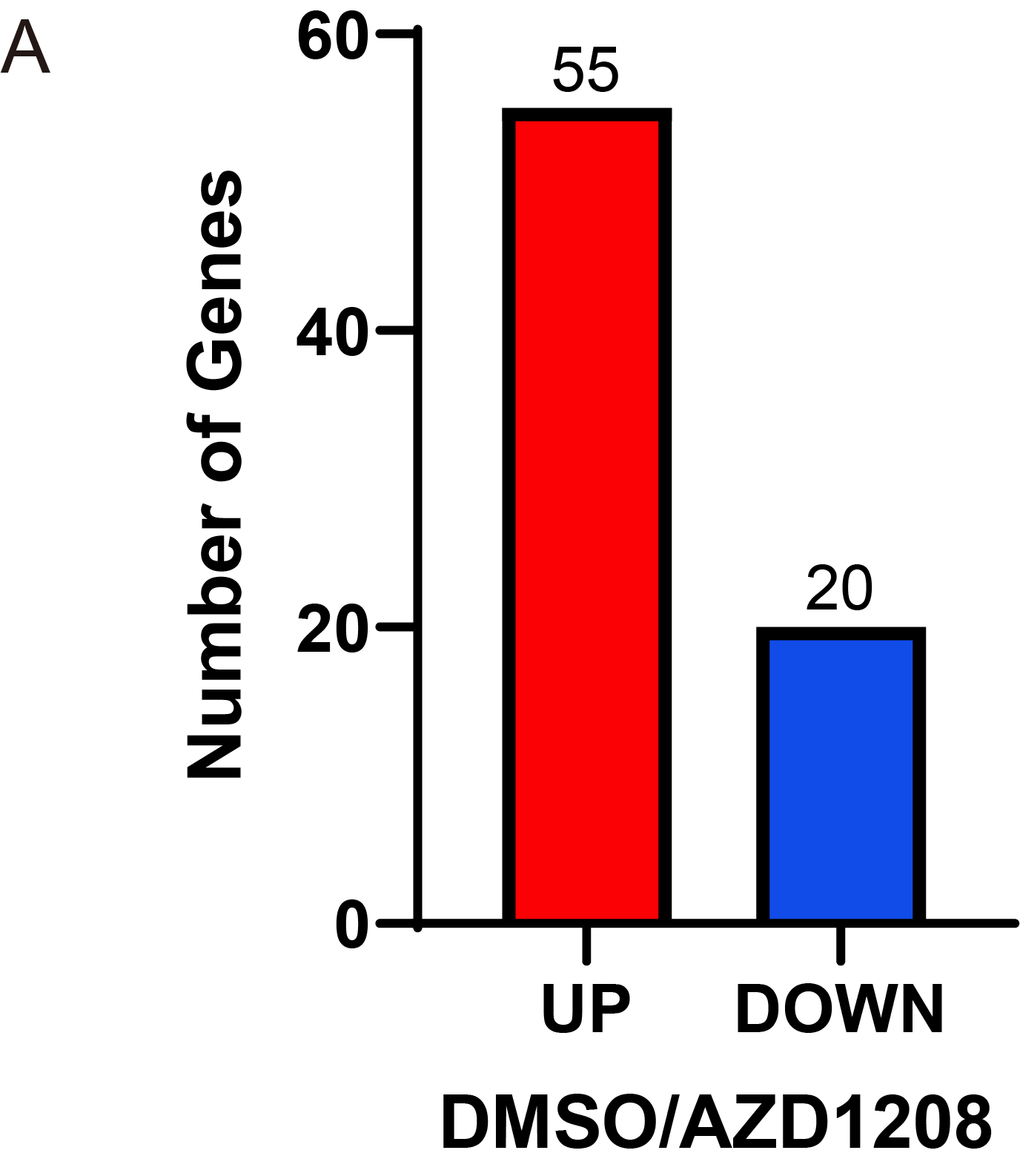

Supplement: Figure S5 — DEGs of CD4+ T cells after inhibiting PIM1 kinase activity by RNA sequencing. [file mbio.01680-25-s0005.tif]

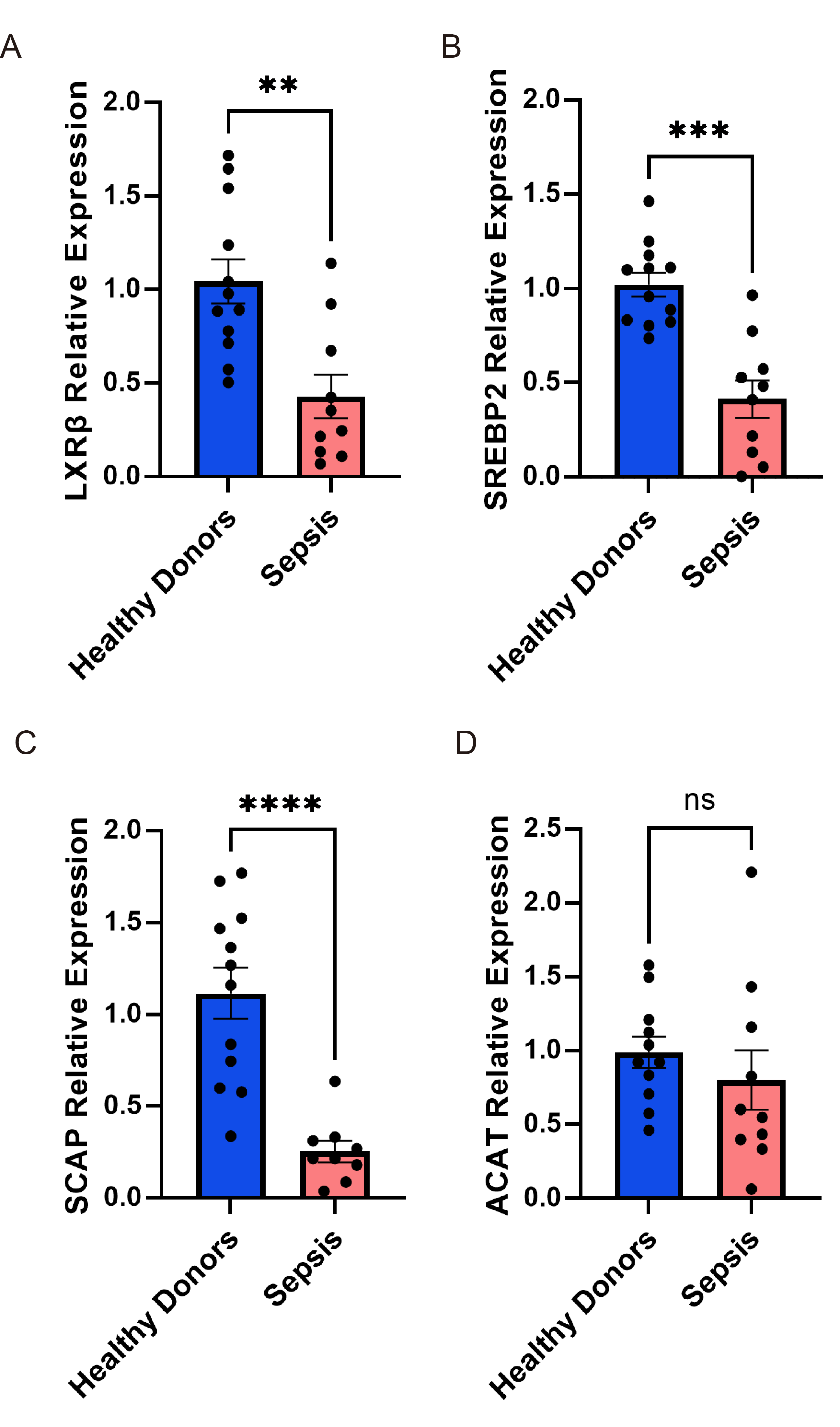

Supplement: Figure S6 — mRNA expression of cholesterol metabolism-related genes in CD4+ T cells from sepsis patients. [file mbio.01680-25-s0006.tif]

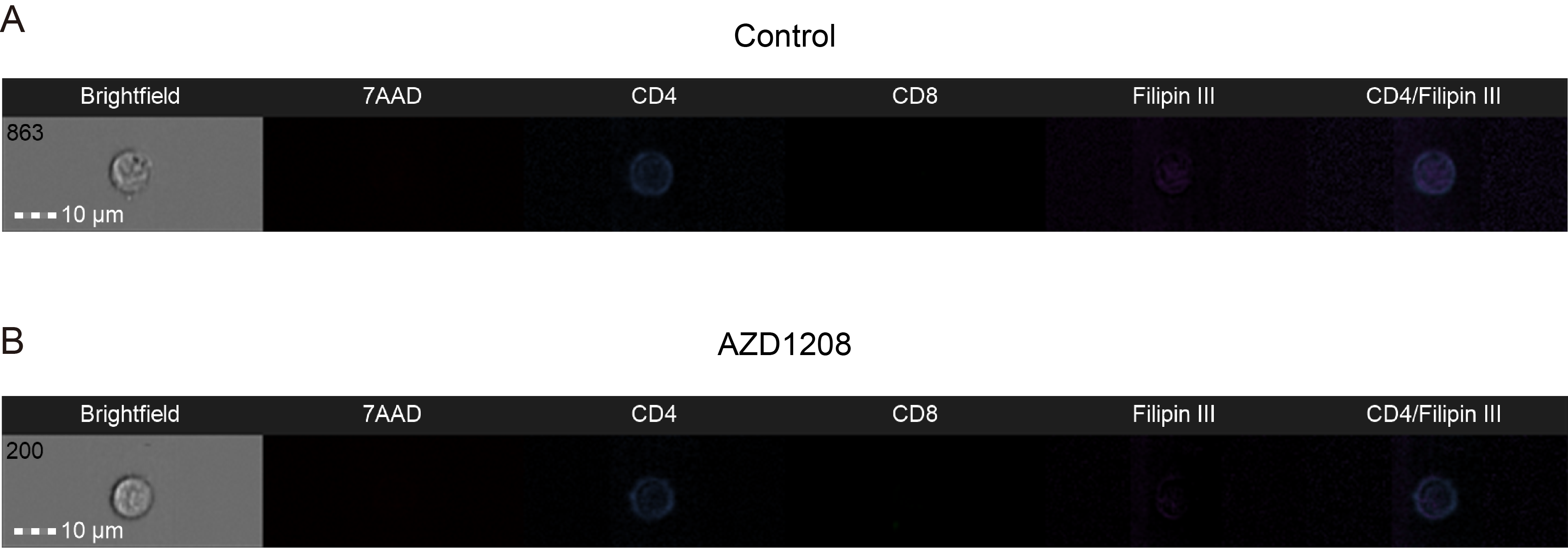

Supplement: Figure S7 — Cholesterol level in CD4+ T cells after inhibiting PIM1 kinase activity. [file mbio.01680-25-s0007.tif]
