# Supplementary material for: PIM1 orchestrates sepsis-associated inflammatory imbalance in CD4+ T cell subsets via cholesterol metabolism
Source: mBio. 2025 Sep 3;16(10):e01680-25. doi: 10.1128/mbio.01680-25 (PMC12505896; doi:10.1128/mbio.01680-25)
Supplement: Legends — Supplemental figure legends. [file mbio.01680-25-s0008.docx]

**Supplemental Figure Legends**

Figure S1. PIM1 expression on CD8^+^ T cells.

A: Gating strategy of PIM1 expression in CD8^+^ T cells from sepsis patients and healthy donors. B and C: Percentage (*p* < 0.0001) and MFI (*p* = 0.0121) of PIM1 protein expression on CD8^+^ T cells from sepsis patients and healthy donors. Healthy Donor (n = 28), Sepsis (n = 29). Data are plotted as mean ± SEM. **p* < 0.05, *****p* < 0.0001.

Figure S2. Viability of CD3^+^ T cells treated with different concentrations of AZD1208.

Figure S3. PIM1 is involved in regulating Th1, Th17, and Treg subsets imbalance.

A: The gating strategy for CD4^+^ T cells; B and C: The expression of CD4^+^IFN-γ^+^ Th1 cells after PIM1 siRNA transfection (n = 7, siCtrl VS siPIM1#1, *p* = 0.0313; siCtrl VS siPIM1#2, *p* = 0.0156); D and E: The expression of CD4^+^IL-17A^+^ Th17 cells after PIM1 siRNA transfection (n = 8, siCtrl VS siPIM1#1, *p* = 0.9453; siCtrl VS siPIM1#2, *p* = 0.0078); F and G: The expression of CD4^+^CD25^+^FoxP3^+^ Treg cells after PIM1 siRNA transfection (n = 7, siCtrl VS siPIM1#1, *p* = 0.0469; siCtrl VS siPIM1#2, *p* = 0.0313). *P* value was calculated by Wilcoxon signed rank test. Data are plotted as mean ± SEM. **p* < 0.05, ***p* < 0.01.

Figure S4. The functional status of CD4⁺ T cell subsets in sepsis.

A and B: Expression of the IL-22 (*p* = 0.0463) in CD4^+^ T cells from sepsis patients and healthy controls. Healthy Donor (n=8), Sepsis (n = 6); C and D: Expression of PD-1(*p* = 0.0190) in CD4^+^ T cells from sepsis patients and healthy controls. Healthy Donor (n = 8), Sepsis (n = 8); E and F: Expression of TIM-3 (*p* = 0.0281) in CD4^+^ T cells from sepsis patients and healthy controls. Healthy Donor (n = 8), Sepsis (n = 8); G and H: Expression of Granzyme B (*p* = 0.3311) in CD4^+^ T cells from sepsis patients and healthy controls. Healthy Donor (n = 11), Sepsis (n = 13); I and J: Expression of Ki-67 (*p* = 0.0048) in CD4^+^ T cells from sepsis patients and healthy controls. Healthy Donor (n = 9), Sepsis (n = 7); K and L: Expression of IFN-γ (*p* = 0.0212) in CD4^+^ T cells from sepsis patients and healthy controls. Healthy Donor (n = 9), Sepsis (n = 11). *P* value was calculated by Mann-Whitney U test. Data are plotted as mean ± SEM. **p* < 0.05, ***p* < 0.01, ns: not significant.

Figure S5. DEGs of CD4^+^ T cells after inhibiting PIM1 kinase activity by RNA sequencing.

Figure S6. The mRNA expression of cholesterol metabolism-related genes in CD4^+^ T cells from sepsis patients.

A, B, C and D: LXRβ (*p* = 0.0026), SREBP2 (*p* = 0.0001), SCAP (*p* < 0.0001) and ACAT1 (*p* = 0.1464) mRNA expression in CD4^+^ T cells from sepsis patients and healthy donors. *P* value was calculated by Mann-Whitney U test. For LXRβ, Healthy Donor (n = 12), Sepsis (n = 10); For SREBP2, Healthy Donor (n = 12), Sepsis (n = 10); For SCAP, Healthy Donor (n = 12), Sepsis (n = 9); For ACAT, Healthy Donor (n = 11), Sepsis (n = 10). Data are plotted as mean ± SEM. ***p* < 0.01, ****p* < 0.001, *****p* < 0.0001, ns: not significant.

Figure S7. The cholesterol level in CD4^+^ T cells after inhibiting PIM1 kinase activity.

A and B: Filipin III staining of intracellular cholesterol in CD4^+^ T cells after inhibiting PIM1 kinase activity examined by imaging flow.
