# Supplementary material for: PIM1 orchestrates sepsis-associated inflammatory imbalance in CD4+ T cell subsets via cholesterol metabolism
Source: mBio. 2025 Sep 3;16(10):e01680-25. doi: 10.1128/mbio.01680-25 (PMC12505896; doi:10.1128/mbio.01680-25)
Supplement: Supplemental Tables — Tables S1 and S2. [file mbio.01680-25-s0009.docx]

| Characteristic | Sepsis (n=67) |
| --- | --- |
| Age-yr | 60.34±15.10 |
| Sex (M/F) | 44/23 |
| APACHE II Score | 14.46±5.71 |
| SOFA Score | 8.32±3.48 |
| CRP, mg/L | 148.32±91.97 |
| PCT, ng/mL | 13.47±22.30 |
| CD4^+^ T cells count, /μl | 397.10±386.03 |
| Lymphocyte count, /μl | 994.12±1042.01 |
| IL-6, pg/ml | 795.59±930.23 |
| IL-10, pg/ml | 52.46±142.23 |
| Primary diseases |  |
| Cholecystitis | 3 |
| Deep cervical abscess | 3 |
| Pneumonia | 7 |
| Intestinal rupture | 5 |
| Ileac pssion | 8 |
| Stomach Perforation | 5 |
| Acute Peritonitis | 10 |
| Acute Pancreatitis | 4 |
| Hepatapostema | 4 |
| Others | 23 |
| Microbe |  |
| Gram-positive | 21 |
| Gram-negative | 18 |
| Fungal | 18 |
| Others | 24 |

**Supplemental Table 1: Baseline characteristics of septic patients included in the**

**study.**

APACHE Ⅱ: Acute Physiology and Chronic Health Evaluation Ⅱ; SOFA: Sequential Organ Failure Assessment; CRP: C-reactive protein; PCT: Procalcitonin, IL-6: Interleukin-6; IL-10: Interleukin-10. ^a^ Data are presented as mean ± SD, or number (%).

**Supplemental Table 2: Characteristics and blood parameters of healthy donors and sepsis patients.**

|  | **Healthy control subjects** | **Patients with sepsis** | ***p* value** |
| --- | --- | --- | --- |
| **Sample size (no.)** | 74 | 67 | NA |
| **Age (years)** | 57.76±9.58 | 60.34±15.10 | NS |
| **Sex (M/F)** | 39/35 | 44/23 | NS |
| **Hemoglobin (g/L)** | 139.69±18.29 | 102.51±26.00 | <0.0001 |
| **Platelets (×10^9^/L)** | 221.91±41.47 | 197.19±122.83 | 0.0069 |
| **WBC (×10^9^/L)** | 5.56±1.24 | 11.50±5.94 | <0.0001 |
| **Neutrophil (%)** | 55.48±6.47 | 82.56±11.36 | <0.0001 |
| **Monocyte (%)** | 6.75±1.24 | 6.05±3.93 | 0.0095 |
| **Lymphocyte (%)** | 34.81±6.68 | 10.46±8.29 | <0.0001 |
| **Eosinophil (%)** | 2.32±1.35 | 0.66±1.23 | <0.0001 |
| **Basophil (%)** | 0.63±0.28 | 0.26±0.20 | <0.0001 |
